# Improving Digital Patient Care: Lessons Learned from Patient-Reported and Expert-Reported Experience Measures for the Clinical Practice of Multidimensional Walking Assessment

**DOI:** 10.3390/brainsci11060786

**Published:** 2021-06-14

**Authors:** Maria Scholz, Rocco Haase, Katrin Trentzsch, Heidi Stölzer-Hutsch, Tjalf Ziemssen

**Affiliations:** Center of Clinical Neuroscience, Department of Neurology, University Hospital Carl-Gustav Carus, Dresden University of Technology, 01307 Dresden, Germany; maria.scholz@uniklinikum-dresden.de (M.S.); Rocco.Haase@uniklinikum-dresden.de (R.H.); Katrin.Trentzsch@uniklinikum-dresden.de (K.T.); Heidi.Stoelzer-Hutsch@uniklinikum-dresden.de (H.S.-H.)

**Keywords:** patient-reported experience measures, expert reported experience measures, walking assessment, multiple sclerosis

## Abstract

Background: Walking assessment (WA) enables meaningful patient mobility assessment. In this context, patient satisfaction with WA can influence assessment compliance and indirectly affect outcomes. One opportunity to assess patient satisfaction is patient-reported and expert-reported experience measures (PREM). Research on PREMs and WA in daily clinical multiple sclerosis (MS) practice does not exist yet. Methods: We surveyed people with MS about their experience and assessed healthcare professionals’ experience via an interview after patients completed WA. Results: Gait parameters were related to perceived difficulty and strain during performance. Less impaired patients perceived the WA to be less difficult and exhausting but were less likely to use WA results for themselves. Men and patients with higher impairment would perform WA more frequently. A good workflow, a fully performed WA with standardized testing, fully functional measurement systems, support and safeguarding by staff in case of falls, direct feedback after the testing, and patients’ motivation are identified by the experts as necessary factors for a successful WA. Conclusions: As patients’ experience has an impact on patients’ outcomes, long-term monitoring of PREMs should become an integral part of the healthcare service to identify and avoid problems early.

## 1. Introduction

Multiple sclerosis (MS) is a neurodegenerative disease characterized by inflammatory-mediated processes throughout the central nervous system resulting in heterogeneous symptomatic presentation and clinical progression with motor, sensory, and cognitive impairments [1]. Among the range of functions that are impaired in people with MS (pwMS), walking is one of the most often affected with significant impact on quality of life as well. With 85% of pwMS being concerned about walking impairments [2], regular gait and balance function monitoring as part of a multidimensional walking assessment is necessary to closely control disease progression and optimize therapy [3,4].

Accurate collection of data reflecting the functionality of pwMS enables a meaningful mobility assessment in addition to standard clinical outcome measures to create a detailed patient profile [5,6,7]. A surrogate marker for high data quality is patient experience whilst receiving care, as satisfied patients are more likely to actively participate in managing their disease, adhere to therapy, and present with higher quality of life [8,9]. Patient satisfaction can be measured by patient surveys, but also a staff interview can contribute to a more patient-centered clinical management [10]. To identify gaps in the healthcare system, patient-reported experience measures (PREMs) are increasingly gaining attention. In comparison to patient-reported outcome measures (PROMs), PREMs do not measure perception of their own disease-related situation with a clinical value, but assess patients’ experience of receiving care. Similar to PROMs, PREMs are used as tools to improve person-centered care, as they reflect the patient’s perspective, increase patient engagement in care, and are associated with better outcomes [11,12,13]. 

PREMs have also been used in research to evaluate the experience of pwMS in a variety of settings. Such studies are designed to determine patients’ level of satisfaction with their disease-modifying therapy [8,14,15,16,17,18,19,20], communication with the physician and nurse [21,22,23,24,25], the healthcare service [26], and diagnostics and management [27], partially including the influence of satisfaction on adherence. Some studies also identified difficulties [28,29,30] and factors for a good healthcare service [31,32] or proposed improvements for communication [23], decision-making [28], or therapy offers [33]. Few studies addressed patients’ experience in combination with gait issues. In five studies, pwMS evaluated different physical therapy services or walking aids. All participants of the evaluation study for a targeted strengthening program were at least satisfied, and 87% adhered to the program [34]. Normann et al. (2012) reported that 64% of respondents ranked the consultation with the physiotherapist as important and very satisfying. Only a little help was needed to fill in the questionnaires [35]. Campbell et al. (2017) were able to define common barriers (mobility, fatigue, continence issues, and transport issues, needing someone to go with the patient) for receiving physiotherapy with their patient survey [29]. Patients in the study of Paul et al. (2014) reported having no or few technical problems with using the computer and the program for web-based physiotherapy. They needed little support and rated the videos as useful [36]. In three studies, patients rated an aid for foot drop and for hip flexion [26,30]. For optimizing the use of the Odstock Dropped Foot Stimulator (ODFS), past and present users of the ODFS were asked via survey about how the stimulator was used, reasons for use, reasons for discontinuing use, encountered problems, and their level of satisfaction with the service. The results showed that 53.3% of the ODFS users used the equipment every day. Indicated problems with using the ODFS included difficulty positioning the electrodes (43.9%), unreliable equipment (39.3%), and skin allergy (22.4%). The explanation and elimination of equipment problems by the staff were rated very positively by 90% [30]. Reasons for acceptance and user satisfaction with lower extremity orthotics in patients with central neurological movement disorders were surveyed in a study by Swinnen et al. (2018) [32]. 86% of the patients were satisfied, but especially for women, lack of safety was the most important aspect for not using the device. Patients also reported comfort, effectiveness, and easy handling to be the most important aspects. The evaluation of the hip flexion assist orthotics revealed a safe and well-tolerated tool with the potential to improve gait performance. The overall mean satisfaction score after 12 weeks was 86.7% [26].

Only one study evaluated patients’ experience with gait diagnostics or monitoring [27]. In the FLOODLIGHT study, participants’ adherence to smartphone- and smartwatch-based assessments to capture MS symptoms including hand motor function, gait and posture, mood, and cognitive impairment was assessed using a patient satisfaction questionnaire. Adherence of pwMS to active testing and passive monitoring with the FLOODLIGHT app was good and showed only a significant small negative correlation with disease duration. The average overall satisfaction score was 74.1 out of 100, showing a significant association with gender. Half of the participants had no problem with any of the active tests, and only one-third would prefer to avoid the 2-min walk test (2MWT). More than 60% of participants would have liked to continue using FLOODLIGHT to understand their MS better and improve their disease management without providing any data feedback, but 90% were interested in seeing the test results [27].

As walking impairments can lead to falls, which in turn worsen gait, effective monitoring is important for pwMS [37]. Higher quality data is generated when patients are satisfied with their monitoring [8] and a holistic concept is applied [7]. This is why PREMs for gait analysis are important indications for a successful implementation of monitoring. Research on PREMs and walking assessment in daily clinical practice does not exist yet.

The aim of this study was to evaluate patients’ experience with and expert opinion on the walking assessment under clinical practice conditions conducted according to the Dresden protocol for multidimensional walking assessment (DMWA) comprising several gait- and balance-related tests and questionnaires [3]. As gait is an important issue in pwMS [2,38], we assumed that patients perceive the walking assessment results to be meaningful. In addition, based on the fact that the DMWA is well-established, we hypothesized that patients are generally satisfied and comfortable with its implementation. We also hypothesized that patients with higher (self-reported) disability have more difficulty in performing and higher strain. We expected no differences between the experience with the implementation of the paper-based and tablet-based questionnaires for self-reported disability. With additional expert interviews, we intended to corroborate the results of the patient survey and to provide information about test difficulties and problems encountered during the walking assessment. The results of both procedures are used to derive recommendations for a successful gait analysis to be implemented into regular patient care in MS care units [39] so that health professionals (HCPs) will be able to perform an assessment that is optimally tailored to the individual patient and clinical practice setting, which will result in increased detail of assessment, adherence, and patient satisfaction.

## 2. Materials and Methods

For recommendations concerning the procedure of a successful walking assessment, we asked pwMS as well as the staff at the Multiple Sclerosis Center (MSC) of the University Hospital Carl Gustav Carus (Dresden, Germany) about their experiences with the walking assessment according to the DMWA [3] via survey and interview. The patient survey and the expert interview were conducted between October 2019 and November 2020 at the MSC.

### 2.1. Patients

To assess feasibility, acceptance, usefulness, and support services during walking assessment, patients were asked to complete an anonymous paper-based satisfaction questionnaire after performing the gait tests during their routine clinic visit at the MSC between October and December 2019. Eligibility criteria for pwMS included the ability to perform walking assessment and a written consent form. In total, we asked 131 patients to participate in the study. Age, gender, MS type, or medication were not criteria for participation. Demographic data (age, gender) and clinical characteristics were collected retrospectively. Clinical characteristics included duration of disease, treatment, and MS subtypes: relapsing–remitting MS (RRMS), primary progressive MS (PPMS), secondary progressive MS (SPMS), and clinically isolated syndrome (CIS). Disability was assessed using Kurtzke’s Expanded Disability Status Scale (EDSS) [40].

### 2.2. Walking Assessment

The walking assessment in its current form has been conducted since 2018. So far, the staff has already gained experience in performing over 5500 tests, with each examination taking about 30 min. The testing procedure was based on the DMWA [3]. 

The GAITRite system from CIR Systems (Franklin, NJ, USA) [41] recorded the Functional Ambulation Profile (FAP) score (scale 0–100), a score for the overall assessment of walking ability. The GAITRite system has a walkway-to-walkway spatial accuracy from ± 1.27 cm and a walkway-to-walkway temporal accuracy from ±1 sample [42] and therefore is a valid and reliable instrument compared to other measurement systems [43,44]. Further mobility and balance parameters (postural sway with eyes open or closed using the Romberg test) were collected with the Mobility Lab from APDM (Portland, OR, USA) [4,45,46]. The Mobility Lab System has also demonstrated a good re-test reliability for pwMS (ICC: 0.85 ± 0.08) [47]. Walking speed is determined by the timed 25-foot walking (T25FW) and the walking endurance by the 2MWT. The two PROMs used for self-assessment of walking ability in pwMS were the Multiple Sclerosis Walking Scale (MSWS-12) and the Early Mobility Impairment Questionnaire (EMIQ). Approximately half of the patients completed the PROMs on a paper-based form, the other half on a tablet-based form. The tablet-based infrastructure is part of the MSDS3D-based documentation system of the MSC [7,48].

### 2.3. The Patient Survey

Patients answered the satisfaction questionnaire in the presence of an HCP after going through the walking assessment with another HCP. This patient-directed survey included three questions on whether conducting the gait analysis was easy, three questions on whether conducting the gait analysis was straining, two questions on whether conducting the gait analysis was comfortable, three questions to rate the usefulness and relevance of the assessment results, one question to evaluate the staff support, and two items assessing the appropriateness of time and frequency of the walking assessment using a scale from 0 (not at all) to 10 (extremely). A score (0–80) for overall satisfaction was formed by adding item values from comfort, support, benefit, sense and use of the results, and acceptance of required time. The original questions are listed in Appendix A.

### 2.4. Expert Interview

In two semi-structured, approximately 90 min long interviews, we asked the staff of the Mobility Center at the MSC about their experiences with performing gait assessment with pwMS. For documentation and later analysis, we used digital recording and note-taking. The selected experts performed an average of 1800 tests per year and have several years of experience. In two sessions with three mobility staff members in each session, they answered a set of predetermined open-ended questions on the categories of feasibility (duration, patient characteristics, technical difficulties, test characteristics, test procedure, environmental factors), utility (result communication, result consequences), necessary support services during assessment (support situation, support type, support goal, unresolved issues), patient acceptance of walking assessment (patient characteristics, situation conditions), and frequency of accomplishment. In addition, HCPs provided recommendations to incorporate a successful gait assessment into daily clinic routine and rated test difficulty on a scale ranging from 0 to 10.

### 2.5. Statistics

Statistical analysis was performed using the SPSS Statistics 27.0 statistical package (IBM, Armonk, NY, USA). Questionnaire data were reported descriptively and expressed as mean or frequency of participants. More detailed information (Standard deviation (SD), confidence interval) can be found in the appendix. The distribution of demographic data is given in frequencies and percentages. Gait characteristics and PROMs were assessed using median, mean, and SD. Correlations between demographic, clinical and gait data, PROMs, and PREMs were calculated with the Kendall’s τ-b correlation coefficient for non-normally distributed data and ordinal data. For Kendall’s Tau-b (τ), levels between 0.1 and 0.3 describe small correlations, levels from 0.3 to 0.5 moderate correlations, and levels above 0.5 large correlations. We used a generalized linear model (GLM) with a Gamma or Tweedie log link function for skewed data and a linear link function for normally distributed data including the factors gender, EDSS, age, medication, disease duration, and type of input (tablet/paper-based). For the corresponding pairwise comparisons, adjustment via Bonferroni correction was applied. The GLM was performed to determine variables influencing PREMs or PROMs. A *p*-value < 0.05 was considered statistically significant.

The evaluation of the expert interviews was conducted by two independent reviewers (M.S., R.H.). First, the experts’ opinions were divided into main categories and then interpreted by the evaluators. The staff’s rating of test difficulty for patients and staff is presented in frequencies.

Ethics Statement: Written informed consent was obtained from the individual(s) for the publication of any potentially identifiable images or data included in this article. The studies involving human participants were reviewed and approved by the Ethics Committee at the Dresden University of Technology. Approval number: EK 224062011.

## 3. Results

### 3.1. Patient Survey

Overall, 80% of pwMS agreed to participate in the survey and 105 patients completed the survey. Table 1 summarizes the characteristics of the study participants who completed the survey.

Mean age was 44 years and ranged from 20 to 74 years. Disease duration ranged from 1 to 53 years and was on average 10 years. Disability status (EDSS) ranged from 1 to 6.5 and had its median at 2. For five patients, we only had PREMs but no further data. Forty-three percent completed the questionnaires via tablet and the other part filled in the paper-based version. Median, means, and SD of gait parameters and PROMs are listed in Table 2.

**Figure 1:**
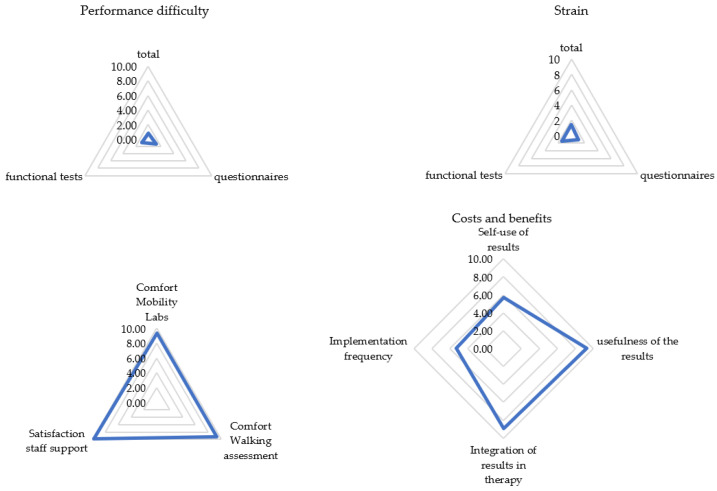
Patient rating for each subcategory (*N* = 105). Categories were rated on a scale from 0 (not at all) to 10 (extremely). Presented are mean values of the categories.

**Figure 2:**
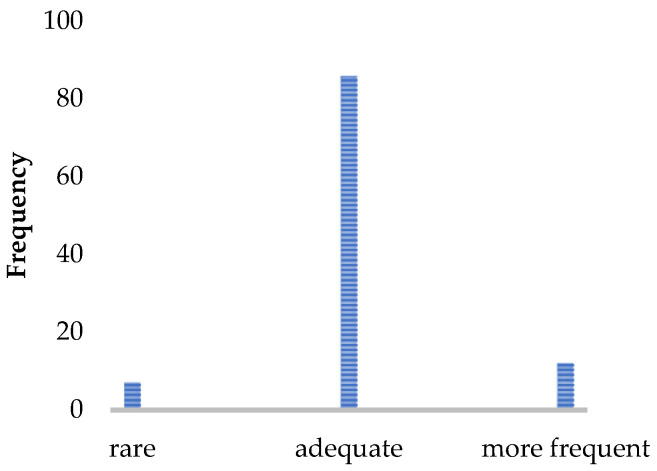
Patient rating of the implementation frequency (*N* = 105). Number of patients who would like to receive the walking assessment less often/more often or find the implementation frequency sufficient.

Visual inspection of the frequency distribution of the satisfaction ratings showed the data to be skewed toward higher satisfaction ratings. In fact, 93% of the PREMs were rated as 1 or 0 (easy; less exhausting), 9 or 10 (useful; convinced; comfortable; supportive; less time-consuming), or 5 (sufficient) with a mean overall satisfaction score of 67 out of 80. Only the question regarding the usage of the results for one’s own evaluation yielded a split result, with 30% not using the results and 34% doing so. See Figure 1 and Figure 2 for all results. Detailed mean values can be found in Appendix B.

Most patients experienced the assessment as easy and not very strenuous. They tended to report the assessment as being comfortable and well-supervised by the staff. The acceptance of the implementation time was very high, and the results of the gait analysis were perceived as very useful and well-incorporated into the therapy management. An implementation per year was considered sufficient by 82% of pwMS.

A higher self-rated walking disability (via MSWS-12 and EMIQ) was associated with higher age (τ = 0.33 and τ = 0.30), longer disease duration (τ = 0.19 and τ = 0.19), higher disease disability (EDSS, τ = 0.63 and τ = 0.60), less walking speed (T25FW, τ = 0.44 and τ = 0.46), less walking endurance (2MWT, τ = −0.41 and τ = −0.43), worse FAP (τ = −0.23/−0.34 and τ = −0.22/−0.34), more balance sway (τ = 0.41/0.24, and τ = 0.44/0.23), and a longer time required for completing the functional tests (τ = 0.20 and τ = 0.22) (Table 3).

Correlations between PREMs and demographic outcomes are summarized in Table 4. Higher age was associated with a higher self-use of the results (τ = 0.24), more confidence that the results will be incorporated into the therapy management, more strain, especially when performing the functional tests, and a higher overall satisfaction (τ = 0.22). A longer disease duration was associated with more difficulties with the functional tests, more strain (τ = 0.20), and less satisfaction with staff support (τ = −0.21).

Correlations for PREMs, clinical outcomes, and PROMs are reported in Table 5 and Table 6. Difficulties in performing the assessment correlated with longer walking time (τ = 0.21), less walking endurance, worse functional ambulation profile, more balance sway (τ_open_ = 0.22 and τ_close_ = 0.26), a longer required implementation time, and higher self-reported gait impairment (τ_MSWS-12_ = 0.21 and τ_EMIQ_ = 0.21). Difficulties in filling out the PROMs were associated with higher disease disability, longer walking time (τ = 0.23), less walking endurance (τ = −0.27), a worse functional ambulation profile for normal walking, more balance sway when eyes open, a longer required implementation time (τ = 0.30), and higher self-reported gait impairment (τ_MSWS-12_ = 0.24 and τ_EMIQ_ = 0.26). Difficulties in performing the functional tests were associated with higher disease disability (τ = 0.26), longer walking time (τ = 0.24), less walking endurance (τ = −0.25), worse FAP, more balance sway (τ = 0.26/0.30), a longer time required for completion (τ = 0.24), and higher self-reported gait impairment (τ_MSWS-12_ = 0.27 and τ_EMIQ_ = 0.26). A higher self-use of the results correlated only with higher disability (τ = 0.27); the confidence that the results will be incorporated into the therapy correlated only with higher disease disability, longer walking time (r = 0.20), and higher self-reported gait impairment (τ_MSWS-12_ = 0.28 and τ_EMIQ_ = 0.23). Higher strain during the assessment correlated with higher disability (τ = 0.37), longer walking time (τ = 0.38), less walking endurance (τ = −0.34), worse FAP (τ_normal_ = −0.24 and τ_dual task_ = −0.24), more balance sway (τ_open_ = 0.27 and τ_close_ = 0.23), a longer required implementation time for the functional tests, and higher self-reported gait impairment (τ_MSWS-12_ = 0.38 and τ_EMIQ_ = 0.42). Higher strain in filling out the PROMs correlated with higher disease disability (τ = 0.30), longer walking time (τ = 0.28), less walking endurance (τ = −0.26), worse FAP (τ_normal_ = −0.20 and τ_dual task_ = −0.23), more balance sway with eyes open, a longer required implementation time (τ = 0.20) and higher self-reported gait impairment (τ_MSWS-12_ = 0.29 and τ_EMIQ_ = 0.30). Higher strain in performing the functional tests was associated with higher disease disability (τ = 0.36), longer walking time (τ = 0.30), less walking endurance (τ = −0.31), worse FAP (τ_normal_ = −0.27 and τ_dual task_ =−0.23), more balance sway (τ_open_ = 0.33 and τ_close_ = 0.25), and higher self-reported gait impairment (τ_MSWS-12_ = 0.43 and τ_EMIQ_ = 0.42). A correlation between higher overall satisfaction and higher EDSS (τ = 0.27) as well as longer walking time as well as higher self-reported gait impairment (τ _MSWS-12_ = 0.28 and τ_EMIQ_ = 0.24) existed.

Variables that determined the self-reported gait impairment are identified in Table 7. The higher the EDSS, the more disabled patients felt regarding their mobility (see Table 8). Gender, age, disease duration, and the method of conducting the questionnaires had no influence on PROMs.

Variables that had an effect on the self-reported experience with the gait assessment are identified in Table 9. Exactly mean values for the subcategories are shown in Table 10.

**Figure 3:**
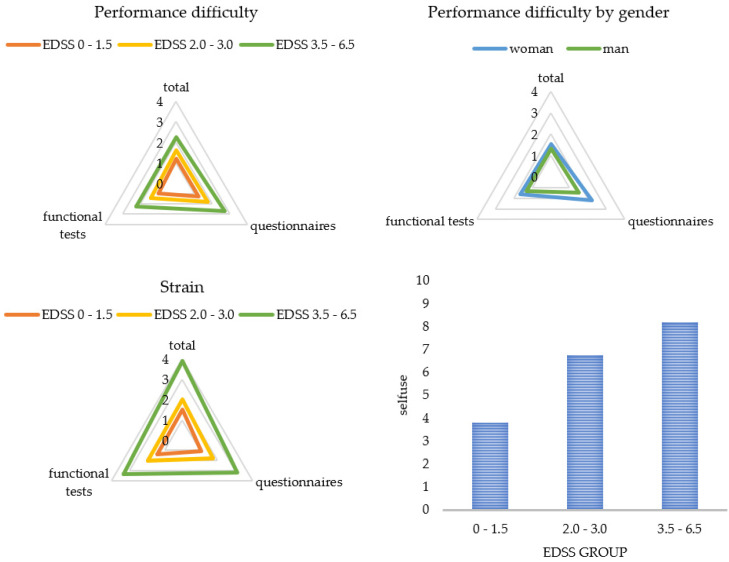
Patient ratings for each subcategory by subgroups. Categories were rated on a scale from 0 (not at all) to 10 (extremely). Presented are mean values of the categories. EDSS = Expanded Disability Status Scale.

**Figure 4:**
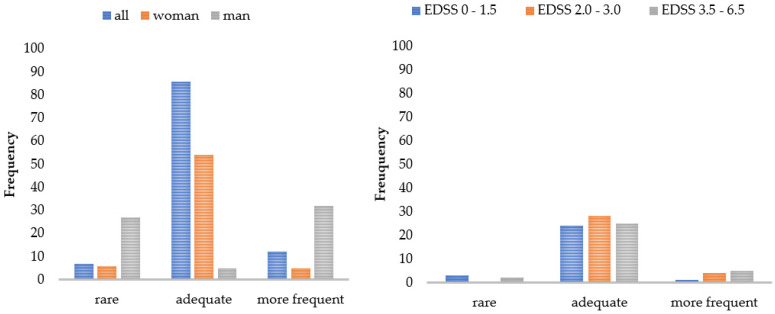
Patient ratings of the implementation frequency by subgroups. Number of patients who would like to receive the walking assessment less/more often or as currently applied. EDSS = Expanded Disability Status Scale.

Figure 3 and Figure 4 display a graphical representation of these results. The higher the disability (EDSS), the more challenging and straining pwMS rated the gait assessment. This applied to the questionnaires as well as to the functional tests. The higher the disability (EDSS), the more the patients utilized the results for themselves and the more often they welcomed the implementation of the gait assessment. Women perceived the functional tests and questionnaires to be more difficult than men did, and men asked for a more frequent implementation compared to women. The results also showed that a higher age predicted less difficulty in performing (B = -0.03), and a longer disease duration was associated with more perceived difficulty (B = 0.03) and strain (B = 0.03) when performing the functional tests. The method of conducting the questionnaires had no influence on PREMs.

### 3.2. Results Expert Interview

Three physical therapists, two students with medical and movement science backgrounds, and one study assistant participated in the interview. The selected experts have performed an average of 1800 tests per year and have one to four years of experience. Asking for necessary factors for describing a successful walking assessment, staff members identified good workflow, fully performed gait analysis with standardized testing, fully functional measurement systems, support for questions and fall prevention, direct feedback after testing from the physician and motivated patients as key characteristics, with transparency and support during testing being the most important.

A smooth workflow was characterized by starting the assessment at the time appointed and finishing within a predefined time period of 30 min. The implementation time depends on the patient and the staff. Better-performing patients with lower disease levels finished the tests faster, but the implementation duration also depended in part on staff’s time needed to prepare the data for evaluation and to give feedback to the patients. Patients knowing the exact examination procedure and time needed enabled a smoother workflow by planning enough time for the visit in their personal schedule, making them less stressed and more motivated to participate in testing. 

**Figure 5:**
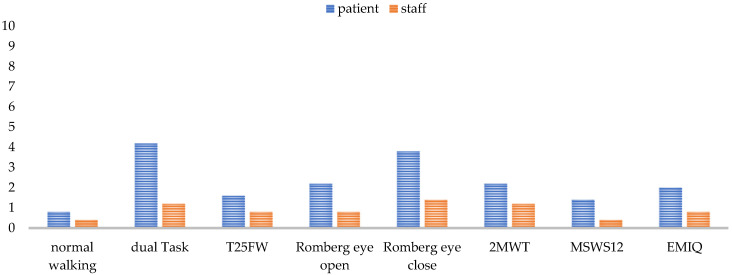
Test difficulty rating for each test from 0 (easy) to 10 (very difficult) for the patient and the staff from staff’s perspective. Normal walking means walking over a mat with a self-selected comfortable walking speed. T25FW = the timed 25-foot walking; 2MWT = 2-min walk test; MSWS-12 = Multiple Sclerosis Walking Scale; EMIQ = Early Mobility Impairment Questionnaire.

The base for obtaining high-quality data in all gait parameters is a fully completed, standardized gait analysis. Annual assessments for SPMS or semi-annual analysis for PPMS were perceived as adequate to detect gait changes in a timely manner. Younger and less impaired patients went through the analysis more easily and with fewer discontinuations. Factors such as the environmental temperature, season, and time of day also seemed to have an influence on performance. In summer, at high temperatures, and in the afternoon, patients experienced more strain. However, experts also reported that time of day was less crucial for patients’ performance than the timing of performing the walking assessment. The more preliminary examinations the patients had already undergone, the more straining the gait assessment was. In general, experts argued that a measurement after a six-hour infusion is too strenuous for the patient and would bias the results, so walking assessment is not performed under such circumstances, whereas the assessment after a one-and-a-half-hour infusion, such as the infusion of natalizumab, is possible. The tests performed and the test sequence determined whether a complete data set could be collected for a patient or not. Each test challenged the patient differently (See Figure 5) and, thus, had an influence on the result of the subsequent tests.

The extent to which a test was a challenge to the patient mostly depended on the patient’s individual performance level. Balance deficits affected all tests, as some tests needed to be omitted, aborted, or secured by the staff due to fall risk. One test that was always feasible to perform was the normal walking (walking over a mat with a self-selected comfortable walking speed) and the T25FW. These two tests were the easiest for the patient to perform and for the staff to monitor. For patients swaying strongly, care must be taken to ensure that they do not leave the mat and thereby interrupt the data recording. Accordingly, functional tests such as the dual task, the Romberg tests, and the 2MWT needed the most support by an HCP (See Figure 5). The questionnaires were also among the more difficult parts of the assessment for staff, as the patients needed some help filling in questionnaires. A problem concerning the 2MWT was the self-selected gait speed, which was perceived differently by the patient depending on their daily form and the semi-standardized instruction. Missing data in patient reports occurred when patients did not recognize that there are still open questions to be answered, or when they did not have time to answer the questions on the tablet in the center due to competing scheduled events.

Equally necessary for obtaining correct data was the use of reliable devices. This included uninterrupted error-free data collection by sensors or tablet, trouble-free data storage, compatibility with all other used systems, and error-free data export. If there was a time delay due to technical disruptions, patients were usually tolerant. Technical reliability in gait analysis still had potential for improvement, although a complete measurement was achieved in about 85% of cases. Considering the use of the tablet, about 20% of the patients experienced difficulties in handling it. In these cases, a pen facilitated the input via touch display. Especially, older patients showed problems in using the touchscreen correctly. Therefore, this group of patients predominantly preferred paper-based questionnaires.

During gait analysis, the staff supported the patients if required to prevent falls and keep the diagnostic process running. For adequate help, staff had to pay careful attention to patients’ feedback. Concerning the questionnaires, help was provided by explaining content-related questions comprehensibly or defining the reference frame more precisely. If patients had problems with handling the technology, the staff demonstrated the optimal way to enter data via tablet. The safety of balance-deficient patients was enhanced by the staff or an additional staff member standing or walking next to the patient during assessment. Especially, supporting while walking on the mat was very important and needed further space to walk next to the patient, even if the patient required an assistive device such as a walker. 

Continuous cross-sectional as well as longitudinal analyses provided information about relevant abnormalities, improvement or decline in gait pattern, and balance. In this way, evidence about the effectiveness of prescribed (medications, medical aids, physiotherapy, rehabilitation) or self-selected interventions (nutrition changes, exercise, workout modifications, and other lifestyle changes) could be gathered. If data showed an overall deterioration exceeding 20% [47] or multiple subtests showed a deterioration of more than 10%, the staff notified the physician according to standard operating procedure. Based on the results of the walking assessment, the physician decided together with the patient on further interventions. Patients used the results for themselves by reviewing their self-evaluation and adapting physiotherapy or training to it. Physicians used the results to adjust and optimize the patient’s therapy, especially symptomatic therapy. For example, the multimodal walking assessment describes clinical response and changes in gait parameters after being treated with fampridine. If a patient was not responding to fampridine, the gait analysis results provided evidence so the patient could discontinue the drug early [49]. Most patients considered the feedback to be sufficient, but patients who did not receive feedback from the physician on the results were less motivated to participate in future examinations and were more dissatisfied with medical care due to the inability to resolve unanswered questions.

Regardless of the previously mentioned aspects, the quality of performance largely depended on the patient’s motivation. The willingness to perform the walking assessment was perceived to be independent of age and was very high at first screenings. Patients who suspected changes in gait as well as those who had the opportunity to improve their physical condition were more motivated than patients who showed no gait abnormalities at all or those who did not show any changes over a long period. Personality structures and mental states also affected motivation. Depressed patients and patients who wanted to avoid negative results were less motivated and hampered the examination, whereas extremely worried patients were willing to do additional examinations. However, experience with previous examinations also influenced patients’ motivation.

## 4. Discussion

In our multimodal study on the experiences of pwMS and HCPs during and after a holistic walking assessment such as the DMWA, we found high acceptance rates and perceived feasibility by pwMS for a systematic half-hour long assessment of walking speed, endurance, balance, and mobility in patients’ everyday living. Correlations would be classified as mild to moderate, which confirms the validity of our used systems [43,44,47], but also illustrates PREMs are not negligible. Patients rated the walking assessment in daily clinical practice as very comfortable (Figure 1), and the high level of overall satisfaction (67 out of 80 score) was consistent with the results for the rating of remote measurements [27]. As gait is one of the most valuable functions for pwMS, patients rated the walking assessment to be very meaningful [38]. As expected, patients without or with minor (self-reported) walking impairments experienced less difficulty in performing the procedure and less strain (Table 10). Walking assessment was also easier for newly diagnosed pwMS (Table 9). These patients were even more motivated and interested (Section 3.2), whereas patients with longer disease duration can develop “disease tiredness”, making testing more difficult for patients, even if they still have a low level of disability. On the other hand, elderly patients experienced less difficulty, which suggests that they were more motivated to perform the assessment, as gait changes for the increasing age arouse additional interest [50]. Men perceived the analysis as less difficult and would perform it more often (see Table 10). One explanation for this may be the fact that men usually develop a shorter disease course with more severe progression [51]. Therefore, they can be more motivated, develop less “disease tiredness”, and, due to the rapid increase in impairment, be more interested in closer monitoring in order to be able to treat at an early stage. Closer monitoring for patients can lead to better analysis of disease progression and faster response to changes. Optimally, mobility will be better preserved. Our study also replicated findings that a lower EDSS was associated with a lower self-reported score [52,53], but the way PROMs were collected, whether via tablet or paper, had no effect on PREMs (Table 10). When patients used the results of the walking assessment, they wanted to monitor the efficacy of therapy and behavioral changes. Elderly patients were especially confronted with gait changes [50], so they use the results more often (τ = 0.24) for control purposes.

Recognizing the experiences and recommendations of our experts, a complete, standardized gait analysis enables the recording of valid and reliable data in order to gain a good overview of the patients’ walking status and the long-term clinical changes. Although the current (semi)annual implementation frequency is sufficient (Figure 2), an extension of diagnostic testing before and after cortisone treatment or continuous measurements in daily life, especially for PPMS, might further improve the granularity of this functional domain of MS. Real-world gait data are especially necessary for detecting the artificial situation of gait, because an assessment in a clinical context always represents only a snapshot of patient’s gait function and is affected by daily fluctuation [54]. Twenty-four-hour analyses with the patients’ daily environmental conditions can represent a more accurate image of the actual gait function. The development of various eHealth approaches is enabling this kind of daily monitoring [55]. For obtaining comparable, valid, and reliable data, it is ideal to start with the whole gait analysis, always at the same time of the year and day and not after a long infusion when the patient is too strained. As the tests strain the patients differentially (Table 5), the test procedure should remain identical each time, and the demand should progressively increase to avoid test abortions and to obtain as many gait parameters as possible from each patient, especially from those with higher impairment. If the testing procedure begins with the most severe tests, there is a risk of not obtaining any results when patients fail the test and are subsequently overstrained for further testing. On the one hand, a standardized test procedure is essential to make the results comparable; on the other hand, an adaptive design of the test protocol according to the patient’s performance spectrum is recommended. For fitter individuals, it would be an option to expand the test protocol from Section 2.1 by adding monopedal hopping and using the 6MWT or running on the treadmill instead of the 2MWT. The use of the 6MWT would be in line with the literature [56], but is, similar to the monopedal hopping, difficult to implement in walking assessment for a patient using a walker. Monopedal hopping provides additional information about lower limb strength in combination with coordination [57,58]. As fitter patients present fatigue symptoms later, endurance testing over a longer distance (6MWT) [56] or with a higher speed (treadmill) [59] is especially recommended for this group of pwMS, as they are more likely to be able to reach the maximum load limit. The 2MWT, together with the eyes-closed balance test, was reported to be the most difficult test (Figure 5) and should be placed at the end of the testing. Normal walking and T25FW were the easiest tests for the patients (Figure 5) and should be positioned at the beginning of the procedure. As the T25FW was the only test that provided data when devices used for the walking assessment failed, care should be taken to ensure that at least this test can be performed. One way to simplify the process for staff is the digitization of individual tests as smartphone-based or web-based applications [55]. Digitization enables patients to perform the tests themselves and saves personnel resources while receiving valid data [60,61].

A disrupted testing procedure would lead to long waiting times and would have a negative effect on patient motivation. Therefore, the staff must be given sufficient time for the examination. Well-trained staff members are essential to ensure a fluid process with minimal waiting time between different tests for the patients. Examination procedure and total examination time need to be transparent for patients to gain their acceptance and to motivate them in doing the tests. This could be implemented via a reminder in advance of the visit using our patient portal [7], reminding the patient about the appointment and the duration of the appointment.

Technical reliability can be guaranteed by regular maintenance of all hard- and software. Reference systems offer an additional possibility to get usable data. When using a camera as a redundancy device, an automatic running and evaluation process simplifies the application. Simple, intuitive, and user-friendly handling leads to better navigation for patients and staff and simplifies the use of technology [55]. To avoid missing data from tabled-based questionnaires (Section 3.2), electronic survey environments should display a summary screen at the end to check whether the patient has completed the questionnaires. Providing paper-based questionnaires to take home for patients who did not have time to fill out the questionnaires or for those who are not able to handle the tablet also allows more data collection. For easier tablet handling, patients should always be given a pen for use.

To secure the patient during the tests, the space for performing must be large enough. Fall prevention in very confined spaces cannot always be ensured by the staff. Nevertheless, to secure walking when staff cannot walk next to the patient, a handle attached to the wall is an additional aid for the patient to hold on to in case of struggling. 

The most important factor for keeping patient acceptance high is feedback (Section 3.2). Feedback should take place immediately after the assessment. Our experts therefore recommended always arranging the physician’s visit after the tests. If a patient requests more details about their disease or detailed examinations of individual bodily functions, the patient can engage in psychoeducational events or additional studies with extended symptomatic diagnostics. As patient motivation can influence the results of the analysis [8], it is necessary to maintain it. Patients need to be reminded repeatedly about the importance of the walking assessment. Moreover, the overall time required and test quantity on one day should be controlled; otherwise, patients’ motivation and thus compliance will decrease (Section 3.2). In order to keep the total test duration low, care must also be taken to ensure that patients are not required to complete tests twice.

PREMs are important indicators of data quality. They should be implemented in long-term monitoring as a criterion for the validity of collected data, as only positive PREMs can guarantee valid data.

Our research is not without limitations. During the implementation of the patient survey, a staff member was always present. It can be assumed that the lack of complete anonymity has led to a result shift towards better ratings [62]. In addition, the exclusion of patients who scheduled limited time for the visit and were therefore under time pressure may have resulted in positively skewed results for the item “acceptance of required time”. Another limitation was the low number of experts. Although the group of experts was mixed, aspects may be missing. Future research should also include physicians’ perspectives. Other important questions that currently remain open are the following: How do examiners cope with documenting walking assessment results? How do they document abortions, eye opening during balance test, and other unexpected events?

## 5. Conclusions

Patients are satisfied with a walking assessment such as the DMWA. Using the results of the assessment, treatments for patients with chronic diseases can be monitored and adjusted. However, outcomes depend on patients’ subjective perceptions and a smooth survey, so PREMs and expert opinion should be used to assess patient satisfaction and improvements for patient care. For responding rapidly to current changes and needs of different patient groups, long-term monitoring of PREMs should become an integral part of the healthcare service.

## Figures and Tables

**Table 1:** Demographic and clinical characteristics of the patients (*N* = 105).

	Variable	Participants, No. (%)
Gender	Female	66 (62.9)
	Male	34 (32.4)
MS subtype	Relapsing–remitting	86 (81.9)
	Primary progressive	7 (6.7)
	Secondary progressive	5 (4.8)
	Clinically isolated syndrome	1 (1.0)
	NA	6 (5.7)
Type of therapy	Aubagio	6 (5.7)
	Avonex	2 (1.9)
	Azatioprin	1 (1.0)
	Copaxone	4 (3.8)
	Gilenya	14 (13.3)
	Interferon	1 (1.0)
	Lemtrada	7 (6.7)
	Ocrevus	23 (21.9)
	Tecfidera	13 (12.4)
	Tysabri	9 (8.6)
	No therapy	19 (18.1)

Abbreviation: MS = multiple sclerosis, NA = not available.

**Table 2:** Patient-reported outcome measures (PROMs) and gait parameters (*N* = 105).

Outcome	Mean ± SD	Median
T25FW (s)	6.38 ± 7.30	4.57
2MWT (m)	151.04 ± 34.88	157.00
MSWS-12 (%)	24.90 ± 27.82	13.00
EMIQ (%)	23.42 ± 25.78	11.00
Duration WA (m:s)	09:22 ± 02:02	08:59
FAP normal	91.78 ± 13.07	96.50
FAP dual task	90.60 ± 13.48	96.00
FAP DIFF	2.5 ± 7.97	0
PSE open (m^2^/s^4^)	0.05 ± 0.08	0.02
PSE closed (m^2^/s^4^)	0.15 ± 0.37	0.03

Duration WA is the time for the implementation of all functional tests without the PROMs. T25FW = the timed 25-foot walking; 2MWT = 2-min walk test, MSWS-12 = Multiple Sclerosis Walking Scale; EMIQ = Early Mobility Impairment Questionnaire, WA = Walking assessment; FAP = Functional Ambulation Profile; FAP DIFF = Differences between normal and dual-task FAP; PSE = Postural Sway Eyes.

**Table 3:** Kendall’s Tau-b (τ) correlation between demographic data, patient-reported outcome measures, and clinical outcomes (*N* = 105).

Outcome	MSWS-12	EMIQ
Age	0.33 **	0.30 **
Disease duration	0.19 *	0.19 *
EDSS	0.63 **	0.60 **
T25FW	0.44 **	0.46 **
2MWT	−0.41 **	−0.43 **
Duration WA	0.20 *	0.22 **
FAP normal	−0.23 **	−0.22 **
FAP dual task	−0.34 **	−0.34 **
FAP DIFF	0.28 **	0.27 **
Postural Sway Eyes open	0.41 **	0.44 **
Postural Sway Eyes closed	0.24 **	0.23 *

* *p* < 0.05; ** *p* < 0.01. Duration WA is the time for the implementation of all functional tests without the patient-reported outcome measures. EDSS = Expanded Disability Status Scale; T25FW = the timed 25-foot walking; 2MWT = 2-min walk test, MSWS-12 = Multiple Sclerosis Walking Scale; EMIQ = Early Mobility Impairment Questionnaire, WA = Walking assessment; FAP = Functional Ambulation Profile; FAP DIFF = Differences between normal and dual-task FAP;.

**Table 4:** Kendall’s Tau-b (τ) correlation between patient-reported experience measures and demographic characteristics (*N* = 105).

PREM	Age	Disease Duration
Performance difficulty total	0.03	0.13
Performance difficulty questionnaires	0.06	0.04
Performance difficulty functional tests	0.05	0.18 *
Self-use of results	0.24 **	0.02
Usefulness of the results	0.08	−0.04
Integration of results in therapy	0.16 *	0.11
Acceptance of required time	0.08	−0.09
Strain total	0.17 *	0.20 *
Strain questionnaires	0.11	0.03
Strain functional tests	0.17 *	0.14
Comfort Mobility Labs	−0.03	0.02
Comfort Walking assessment	0.07	0.03
Staff support performance	−0.14	−0.21 *
Rating of implementation frequency	0.15	−0.02
Overall Satisfaction	0.22 **	−0.01

* *p* < 0.05; ** *p* < 0.01. Overall satisfaction was formed by adding item values from comfort; support; benefit, sense, and use of the results; acceptance of required time.

**Table 5:** Kendall’s Tau-b (τ) correlation between patient-reported experienced measures and clinical outcomes (*N* = 105).

Patient-Reported Experience Measures	EDSS	T25FW	2MWT	Duration WA	FAP Normal	FAP Dual Task	FAP DIFF	PSE Open	PSE Closed
PD total	0.11	0.21 **	−0.19 *	0.19 *	−0.17 *	−0.20 *	0.13	0.22 *	0.26 **
PD questionnaires	0.17 *	0.23 **	−0.27 *	0.30 **	−0.13	−0.17 *	0.18 *	0.16 *	0.10
PD functional tests	0.26 *	0.24 *	−0.25 *	0.24 **	−0.18 *	−0.29 **	0.23 **	0.26 *	0.30 *
Self-use of R	0.27 **	0.24	−0.12	0.15	-0,04	−0.11	0.08	0.08	0
Usefulness of the R	0.13	0.06	−0.34	0.07	−0.08	−0.04	0	0.02	−0.08
Integration of R in therapy	0.18 *	0.20 *	−0.02	0.05	−0.12	−0.12	0.07	−0.04	−0.04
Acceptance required time	0.10	0.08	0.11	−0.06	−0.12	−0.02	−0.14	−0.03	−0.18 *
S total	0.37 **	0.38 **	−0.34 **	0.19 *	−0.24 **	−0.24 **	0.14	0.27 **	0.23 **
S questionnaires	0.30 **	0.28 **	−0.26 **	0.20 *	−0.20 *	−0.23 *	0.18 *	0.19 *	0.14
S functional tests	0.36 **	0.30 **	−0.31 **	0.13	−0.27 **	−0.23 *	0.09	0.33 **	0.25 **
Comfort Mobility Labs	0.03	0.03	−0.05	−0.17 *	−0.01	−0.09	0.02	0.08	−0.09
Comfort WA	−0.02	−0.03	0.01	−0.04	−0.08	0.01	−0.07	0.09	−0.01
staff support performance	−0.06	−0.06	0.06	−0.01	0.05	0.05	−0.03	−0.04	−0.04
Rating of IF	0.15	0.11	−0.02	0.04	−0.06	−0.06	−0.04	0.13	0
Overall Satisfaction	0.27 **	0.15 *	−0.08	0.11	−0.10	−0.14	0.06	−0.04	0.09

* *p* < 0.05; ** *p* < 0.01. Overall satisfaction was formed by adding item values from comfort; support; benefit, sense, and use of the results; acceptance of required time. PD = Performance difficulty; R = results; S = Strain; WA = Walking assessment; IF = implementation frequency; EDSS = Expanded Disability Status Scale; T25FW = the timed 25-foot walking; 2MWT = 2-min walk test, FAP = Functional Ambulation Profile; FAP DIFF = Differences between normal and dual-task FAP; PSE = Postural Sway Eyes.

**Table 6:** Kendall’s Tau-b (τ) correlation between patient-reported outcome measures and patient-experienced outcome measures (*N* = 105).

Patient-Reported Experience Measures	MSWS-12	EMIQ
Performance difficulty total	0.21 *	0.21 *
Performance difficulty questionnaires	0.24 **	0.26 **
Performance difficulty functional tests	0.27 **	0.26 **
Self-use of results	0.28 **	0.23 **
Usefulness of the results	0.12	0.15
Integration of results in therapy	0.11	0.04
Acceptance of required time	0.05	0.09
Strain total	0.38 **	0.42 **
Strain questionnaires	0.29 **	0.30 **
Strain functional tests	0.43 **	0.42 **
Comfort Mobility Labs	0.04	0.07
Comfort Walking assessment	0.03	0.01
Staff support performance	−0.06	−0.07
Rating of implementation frequency	0.21 *	0.23 *
Overall Satisfaction	0.28 **	0.24 **

* *p* < 0.05; ** *p* < 0.01. Overall satisfaction was formed by adding item values from comfort; support; benefit, sense, and use of the results; acceptance of required time. MSWS-12 = Multiple Sclerosis Walking Scale; EMIQ = Early Mobility Impairment Questionnaire.

**Table 7:** Model effects for patient-reported outcome measures and demographic or clinical data.

	Factors	Patient-Reported Outcome Measure	
		MSWS-12 (*n* = 88)	EMIQ (*n* = 90)
*p*-value	Gender	0.206	0.225
	EDSS	0.000	0.000
	Via paper/tablet	0.907	0.253
	Age	0.855	0.767
	Disease duration	0.055	0.136

We used a Generalized Linear Model with Tweedie Log Link Function (factors: gender, EDSS, age, Medication, disease duration, and tablet/paper-based). *p* < 0.05 is significant. MSWS-12 = Multiple Sclerosis Walking Scale; EMIQ = Early Mobility Impairment Questionnaire; EDSS = Expanded Disability Status Scale.

**Table 8:** Mean values of patient-reported outcome measures for subcategories with a significant effect.

Patient-Reported Outcome Measure	Expanded Disability Status Scale		
	0–1.5	2.0–3.0	3.5–6.5
MSWS-12 (*n* = 88)	2.47 ^AC^	19.21 ^AB^	44.31 ^BC^
EMIQ (*n* = 90)	3.88 ^A^	18.27 ^A^	47.30 ^A^

Results from a Generalized Linear Model approach with Bonferroni correction for pairwise comparisons. A higher value symbolizes higher self-reported impairment. MSWS-12 = Multiple Sclerosis Walking Scale; EMIQ = Early Mobility Impairment Questionnaire. ^A.^ *p* < 0.01. ^B.^ *p* < 0.05. ^C.^ *p* < 0.01.

**Table 9:** Model effects for patient-reported experience measures, demographic, and clinical data (*N* = 93).

Patient-Experienced Reported Outcome Experience Measures	*n*	*p*-Value				
		Gender	EDSS	PROMs via Paper/Tablet	Age	Disease Duration
Performance difficulty total	88	0.250	0.005	0.344	0.022	0.003
Performance difficulty questionnaires	91	0.013	0.001	0.062	0.047	0.442
Performance difficulty functional tests	93	0.042	0.000		0.001	0.000
Self-use of results	92	0.350	0.021	0.837	0.600	0.970
Usefulness of the results	93	0.082	0.136	0.529	0.432	0.772
Integration of results in therapy	92	0.865	0.497	0.159	0.844	0.845
Acceptance ofrequired time	93	0.133	0.537	0.282	0.415	0.489
Strain total	93	0.862	0.000	0.382	0.490	0.003
Strain questionnaires	91	0.092	0.000	0.320	0.167	0.630
Strain functional tests	93	0.901	0.000		0.597	0.002
Comfort Mobility Labs	93	0.633	0.671		0.608	0.869
Comfort Walking assessment	92	0.096	0.527	0.542	0.558	0.793
Staff support	92	0.367	0.527	0.728	0.737	0.475
Rating of implementation frequency	93	0.007	0.024	0.485	0.695	0.854

We used Generalized Linear Models with Gamma log and linear link function to rate implementation frequency, factors: gender, EDSS, age, dedication, disease duration, and tablet/paper-based. *p* < 0.05 is considered significant. PREM = patient-reported experience measures; PROM = patient-reported outcome measure; the factor “via paper/tablet” was omitted for PREMs that did not refer to PROMs implementations. EDSS = Expanded Disability Status Scale.

**Table 10:** Mean values of patient-reported experience measures for subcategories with a significant effect (*N* = 93).

Patient-Reported Experience Measures	Gender		EDSS			PROMs via	
	Female	Male	0–1.5	2.0–3.0	3.5–6.5	Paper	Tablet
Performance difficulty total	1.52	1.31	1.03 ^A^	1.36	2.00 ^A^	1.32	1.50
Performance difficulty questionnaires	2.67 ^B^	1.82 ^B^	1.54 ^A^	2.13 ^C^	3.27 ^AC^	1.94	2.51
Performance difficulty functional tests	1.86 ^B^	1.47 ^B^	1.14 ^AB^	1.58 ^BD^	2.51 ^AD^		
Self-use of results	5.43	6.53	3.82 ^BC^	6.75 ^B^	8.20 ^C^	6.07	5.84
Strain total	1.70	1.65	1.13 ^A^	1.44 ^D^	2.89 ^AD^	1.78	1.58
Strain questionnaires	2.39	1.92	1.27 ^A^	2.08 ^A^	3.72 ^A^	2.01	2.28
Strain functional tests	2.02	2.06	1.36 ^AB^	1.93 ^C^	3.22 ^AC^		
Rating of implementation frequency	5.21 ^A^	6.17 ^A^	4.91 ^A^	5.99 ^A^	6.17	5.55	5.83

Results from a Generalized Linear Model approach with Bonferroni correction for pairwise comparisons. Categories were rated on a scale from 0 (not at all) to 10 (extremely). PROM = patient-reported outcome measures; EDSS = Expanded Disability Status Scale. ^A.^ *p* < 0.01. ^B.^ *p* < 0.05. ^C.^ *p* < 0.05. ^D.^ *p* < 0.01.

## Data Availability

The data presented in this study are available on reasonable request from the corresponding author.

## Appendix A

Questions from the walking assessment survey

1. How easy or difficult would you rate …? (0 = easy,10 = difficult)
  - the total walking assessment procedure
  - completing the questionnaires (EMIQ und MSWS-12)
  - completing the function tests
2. To which extent do you use the results for your own review? (0 = not at all, 10 = always)
3. How useful do you think is incorporating the results into your progress monitoring? (0 = not useful, 10 = useful)
4. How convinced are you that the results will be used for your disease progression? (0 = not at all convinced; 10 = convinced)
5. How do you rate the time required to perform the walking assessment? (0 = too time-consuming; 10 = appropriate)
6. How exhausting do you experience …?(0 = not exhausting; 10 = exhausting)
  - the total walking assessment
  - completing the questionnaires (EMIQ und MSWS-12)
  - completing the function tests
7. How do you rate the wearing comfort of the Mobility Lab? (0 = very uncomfortable; 10 = comfortable)
8. How do you rate the comfort of the walking assessment? (0 = very uncomfortable; 10 = comfortable)
9. How do you rate the support and explanation provided by the study staff? (0 = bad; 10 = very good)
10. Do you think the walking assessment once a year is sufficient (if no relapses occur)?
  - Walking assessment should be more frequent []
  - Frequency is sufficient []
  - Walking assessment should be less frequent []

## Appendix B

**Table A1:** Detailed description of the patient-reported experience measures.

Patient-Reported Experience Measures	MEAN ± SD	CI95
Performance difficulty total	0.78 ± 1.74	0.42–1.14
Performance difficulty questionnaires	1.07 ± 2.06	0.72–1.43
Performance difficulty functional tests	0.71 ± 2.03	0.37–1.04
Self-use of results	5.59 ± 4.25	4.76–6.43
Usefulness of the results	9.30 ± 1.53	8.99–9.60
Integration of results in therapy	8.92 ± 1.72	8.58–9.26
Acceptance required time	9.07 ± 1.89	8.70–9.44
Strain total	1.46 ± 2.38	0.92–1.80
Strain questionnaires	1.04 ± 1.92	0.67–1.42
Strain functional tests	1.43 ± 2.42	0.83–1.70
Comfort Mobility Labs	9.45 ± 1.21	9.21–9.68
Comfort Walking assessment	9.36 ± 1.18	9.12–9.59
Staff support performance	9.84 ± 0.54	9.73–9.95
Rating of implementation frequency	5.25 ± 2.17	4.82–5.67

SD = Standard deviation; CI = confidence interval
